# Assessment of liquefaction resistance of soil with fines using cyclic hollow cylinder testing

**DOI:** 10.1371/journal.pone.0329109

**Published:** 2025-08-06

**Authors:** Jungang Liu, Liang Feng, Yi Zhang, Geng Chen

**Affiliations:** 1 Department of Civil Engineering, College of Civil Engineering and Architecture at the Hubei Polytechnic University, Huangshi, Hubei,; 2 Center for Geotechnical Engineering Science, University of Colorado Denver, Aurora, Colorado,; 3 College of Civil Engineering, Guilin University of Technology, Guilin, China; 4 Department of Civil Engineering, University of Colorado Denver, Research Member, Center for Geotechnical Engineering Science, Aurora, Colorado; Institute of Disaster Prevention, CHINA

## Abstract

Soil liquefaction is a devastating effect of earthquakes. It occurs when saturated granular soils lose their shear strength because of a sudden increase in pore water pressure under dynamic loads. Over the last six decades, considerable focus has been placed on understanding the mechanisms and phenomena associated with liquefaction, making it a critical area of research. Evaluating soil liquefaction accurately is crucial for maintaining the seismic safety of construction. To investigate how fines content affects soil liquefaction resistance under cyclic simple shear loads and gradual principal stress rotation, a series of cyclic hollow cylinder tests (CHCT) were carried out under isotropic consolidation and undrained conditions. The experiments were conducted using medium Monterey No. 0/30 Sand (MS), where five varying percentages of fine content were analyzed under two confining pressures (σ_3_’ = 103 kPa and 206 kPa) and at two relative densities (Dr = 30%, 45% and 60%). The results of CHCT tests contributed to the development of liquefaction-potential evaluation curves. The findings demonstrated that increasing the acceptable fines content up to 15% reduces liquefaction resistance. However, when fines content exceeds 15%, further increases lead to enhanced liquefaction resistance. Based on all laboratory test results, back propagation neural network (BPNN) was applied to predict cyclic stress ratios leading to initial liquefaction after cyclic loading cycles. The BPNN model can give superior precision with mean absolute percentage error (MAPE) values of 1.05%, and also can help engineering better understand liquefaction potential of soil samples with different fines content.

## 1. Introduction

The earthquakes in Alaska, USA, and Niigata, Japan, which caused soil liquefaction and resulting damages, highlighted the need for geotechnical engineering research focused on earthquake-related soil liquefaction. In the past sixty years, the geotechnical earthquake engineering community has dedicated significant research efforts to understanding the liquefaction potential of different soil types. A reliable and accurate method for predicting how fine content influences soil liquefaction resistance is crucial for evaluating the seismic safety of buildings and earth structures.

Hollow cylinder (HC) test apparatus is ideal for evaluating the liquefaction potential of different soils. It closely simulates initial field stresses and loading conditions and provides near-uniform stress and strain distributions. Besides, its gradual rotation of principal stresses can closely simulate earthquake-induced dynamic simple shear loading.

Many previous laboratory and field studies have examined how higher fines content affects the liquefaction resistance of soils. The research suggested that raising the allowable sand content affects liquefaction resistance, with critical (or threshold) fine content serving as a dividing limit for this relationship (Chang et al., 1982 [1]; Chang, 1990 [2]).

Field studies conducted after significant earthquakes have produced inconsistent findings regarding the influence of fine content on sands’ liquefaction resistance. Observations of soil behavior during seismic events have shown that sands with a higher fine content are generally less prone to liquefaction.

Okashi (1970) [3] found that sands containing less than 10 percent fine particles were more prone to liquefaction during the 1964 Niigata earthquake in Japan. Fei (1991) [4] noted that during the 1976 Tangshan earthquake, soils with higher fine content exhibited increased liquefaction resistance. Furthermore, a study by Tokimatsu and Yoshimi (1983) [5] on 17 global earthquakes found that half of the liquefied soil samples contained less than 5 percent fines. Studies on earthquake case histories show that sands containing over 10 percent fines have greater liquefaction resistance than clean sands with a similar Standard Penetration Test (SPT) blow count.

Tatsuoka et al. (1980) [6] showed that some field-based methods could determine soil liquefaction potential and calculate fines in the samples. These field methods use SPT blow counts and CPT readings. Seed and Idriss (1971) [7] indicated a simplified procedure for evaluating soil liquefaction potential based on SPT case histories. Seed et al. (1985) [8] demonstrated that the resistance to liquefaction rises with higher fine content, as illustrated by the cyclic stress ratio (CSR) curves compared to corrected SPT blow count.

**Fig 1:**
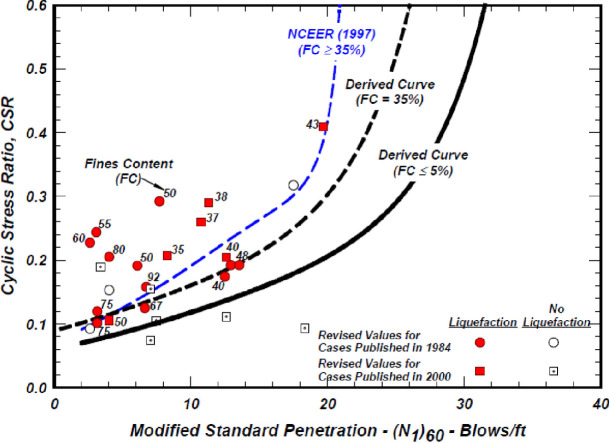
Case histories from the SPT of cohesionless soils with a fine content (FC) of 35% or greater include the NCEER Workshop (1997) curve, along with the suggested curves for both clean sand and FC = 35% for a magnitude of 7½ and a vertical effective stress of 1 atm. (Idriss and Boulanger, 2004 [9]).

Idriss and Boulanger (2004) [9] discovered through SPT case histories of soils that specimens with a fines content (FC) of 35% or more required a higher cyclic stress ratio to achieve liquefaction at a specified blow count. In Fig 1, the curves show that the CSR rises with a higher fine content, while maintaining the same corrected (N_1_)_60_.

Many researchers have explored how fine content influences liquefaction resistance through laboratory studies. A comprehensive triaxial test program of over 400 cyclic triaxial tests (CTT) on the influence of increasing fines content and fines plasticity using the mixtures of a graded Denver sand and fines of the mixture of high plastic Yazoo clay and Vicksburg silt from Mississippi to form fines of different plasticity (Chang, 1990 [2]; H.C. Liu, 1992 [10]; H.C. Liu & Chang, 1994 [11]). The research indicated that the effect of higher fine content on sand’s liquefaction resistance is significantly greater than that of plasticity. Research findings showed that as percentage of fines in the sand increases, the liquefaction resistance first decreases, reaching a minimum at a critical (or threshold) fine content before increasing again. The influence of fine plasticity is much less important. H.C. Liu, 1992 [10] utilized Chang’s database to determine that clean sand with a consistent overall void ratio exhibited the greatest liquefaction resistance. Introducing fines with the same plasticity index to the clean sand, while keeping the clean sand void ratio constant, resulted in a decrease in liquefaction resistance. The decline persists until the fines content hits a crucial threshold of about 20 to 30%, after which the trend changes.

Bray and Sanico (2006) [12] demonstrated that sandy locations with a substantial fine content may be vulnerable to liquefaction. This conclusion was drawn from data collected at various sites with fine-grained soils that experienced ground failure during the 1999 Kocaeli Earthquake and the outcomes of cyclic triaxial tests conducted on samples from specific study sites.

Ueng, Sun, Wen and Chen (2013) [13] demonstrated that utilizing FC400 (which passes through the No. 400 sieve) provides a more effective method for examining the influence of fines on the liquefaction resistance of soil compared to FC200 (which passes through the No. 200 sieve). Laboratory tests conducted in cycles revealed that the cyclic resistance ratio (CRR) declines as the fine content increases. This trend persists until around 20% of the fine content is reached, which aligns with Chang’s observations.

In a study conducted by Park and Kim (2013) [14], loose, medium, and dense specimens containing 10% fines were subjected to undrained cyclic triaxial tests. The findings showed that silty sands had reduced liquefaction resistance with an increase in the plasticity of the fine particles.Medium and dense soil samples’ liquefaction resistance significantly decreased with increased plasticity, whereas the loose samples exhibited only a slight reduction.

Eseller-Bayat et al. (2019) [15] conducted cyclic simple shear tests to investigate how factors such as plasticity, fines content, relative density, and cyclic stress ratio (CSR) influence the liquefaction resistance of sands with low fines content (up to 10%). The research revealed that clean sand showed higher liquefaction resistance than sands containing both plastic and non-plastic fines, even when accounting for equivalent relative densities.

Hudson et al. (2024) [16] observed that the impact of soil plasticity on model residuals was influenced by cone penetration testing (CPT) and the soil fines content. Their model can be utilized to estimate fines content (FC) when CPT data is accessible, but FC has not been measured. However, these estimates should be regarded as having an inherent level of uncertainty.

Ecemis N et al. (2024) [17] found that a new correlation was proposed between the cone penetration resistance and normalized shear wave velocity V_s1_ using the soil-type index Ic representing the fine content. In the cyclic direct simple shear test, the liquefaction resistance reduces with a decrease in Ic from 2.9 to 1.3. The trend changes for V_s1_ less than 170 m/s and (CRR)_7.5_ increases with a reduction in Ic from 1.3 to 2.9. This represents a positive influence of soil fines on liquefaction resistance for V_s1_ more than 170 m/s.

This study intends to implement CHCT to assess the liquefaction potential of sand with acceptable levels of delicate content. The goal is to develop a BPNN model that can predict the liquefaction resistance of soils with varying percentages of this delicate content.

## 2. Test materials

This study employs CTT and CHCT to explore liquefaction resistance of soil mixtures made from Monterey 0/30 Sand (MS) and Leyden Clay (LC) obtained from on public lands within an approximate 10-mile radius of Golden, Colorado. The clay content varies between five and thirty-five percent, with the Leyden clay having a plasticity index of twenty percent (as shown in Table 3).

Monterey 0/30 Sand, a classic clean soil sourced from on public lands within an approximate 20-mile radius of Monterey, California, has been subjected to various laboratory tests. The tasks involved in this process were to identify (1) the minimum and maximum unit weights; (2) specific gravity; (3) friction angle; (4) the relationship between dry density and moisture content, which was established through Standard Proctor Compaction tests to determine the maximum dry density and optimum moisture content. All tests were conducted according to the applicable ASTM International standards designations D3080-04 (2012) [18], D698 (2012b) [19] and D854 (2014) [20]. Table 1 presents the findings, indicating that Monterey 0/30 Sand is categorized as SP according to the Unified Soil Classification System.

**Table 1:** Properties of Monterey Sand.

Monterey Sand (MS)	
Maximum Unit Weight (kg/m^3^)	1695
Minimum unit weight (kg/m^3^)	1469
Specific Gravity:	2.65
Friction Angle (degrees)	37

The Leyden Clay, used in this study as a fine material, was sourced from Pioneer Sand Company near Golden, Colorado. The laboratory tests conducted on the Leyden Clay included atterberg limits, specific gravity, and the standard proctor compaction characteristics. The tests were conducted following ASTM International standards designations D3080-04 (2012) [18], D698 (2012b) [19] and D854 (2014) [20]. The findings are presented in Table 2. Based on the Unified Soil Classification System (USCS), Leyden Clay is categorized as medium plasticity clay (CL).

**Table 2:** Properties of Leyden Clay.

Leyden Clay (CL)			
Liquid Limit (%)	Plastic Limit (%)		Plasticity Index (%)
40	20		20
Optimum Moisture Content (%):		17	
Specific Gravity:		2.67	
Maximum Dry Unit Weight (kg/m^3^):		1778	

**Table 3:** Uniformity coefficients and coefficients of curvature values for five soil samples with fines content.

Sample	FC%	d_60_	d_30_	d_10_	C_u_	C_c_
1	5	0.5	0.4	0.27	1.85	1.19
2	10	0.5	0.4	0.16	3.13	2.00
3	15	0.48	0.28	0.11	4.36	1.48
4	25	0.45	0.2	0.094	4.79	0.95
5	35	0.44	0.15	0.07	6.29	0.73

**Fig 2:**
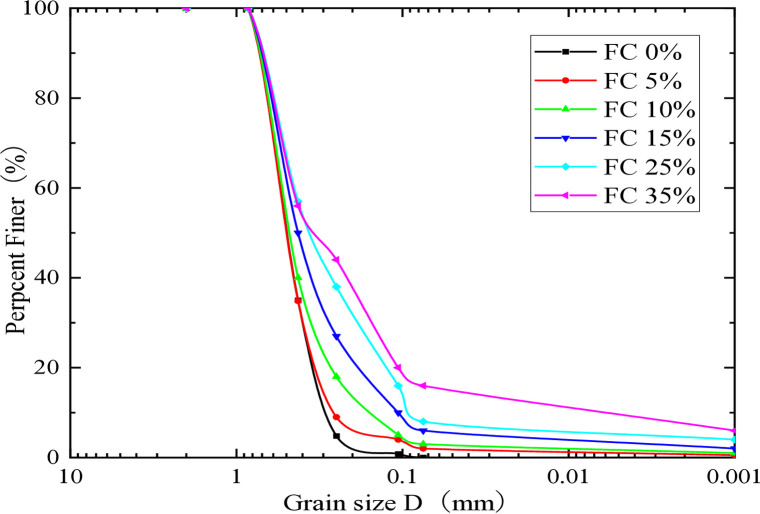
Distribution of grain sizes in the soil samples utilized.

Early laboratory research highlights the significant role that sample preparation techniques play in determining the liquefaction resistance of soils. A wet-tamping method applied to moist soils resulted in the creation of consistent and high-quality saturated samples for cyclic hollow cylinder testing. A total of thirty-seven CHCTs were conducted utilizing a blend of MS and LC, incorporating fine material percentages of 5%, 10%, 15%, 25%, and 35%. The Leyden clay, known for its plasticity index of 20%, was pivotal in these tests.The grain size distribution curves for soils with different fine content percentages are illustrated in Fig 2.

## 3. Test apparatus and high-quality samples

This research utilizes the Hollow Cylinder Axial/Torsional cell at the University of Colorado Denver (UCD) to explore how fine particles influence soil liquefaction resistance. All cyclic hollow cylinder specimens have an inner diameter of 8 inches (203.2 mm), an outer diameter of 10 inches (254 mm), and a height of 10 inches (254 mm). They are prepared under two effective confining pressures (σ_3_’) of 103 kPa and 207 kPa and tested at a frequency of 0.5 Hz, in accordance with the ASTM D5311/D5311M-13 standard [21].

This study uses a wet tamping method to prepare high-quality samples for cyclic hollow cylinder tests on saturated samples prepared from clean parent sand with different percentages of Leyden clay fines. Test samples of a mixture of MS and LC are prepared to obtain the same void ratios as the parent sand at relative densities of 30%, 45%, and 60%, respectively.

To prepare the samples for 37 CHCTs, the MS is blended with Leyden Clay fine at percentages of 5%, 10%, 15%, 25%, and 35%. The samples were produced at three relative densities: 30%, 45%, and 60%. They were subjected to effective confining pressures of 103 kPa and 207 kPa, along with four cyclic stress ratios of 0.2, 0.25, 0.3, and 0.4.

## 4. Cyclic hollow cylinder test

**Fig 3:**
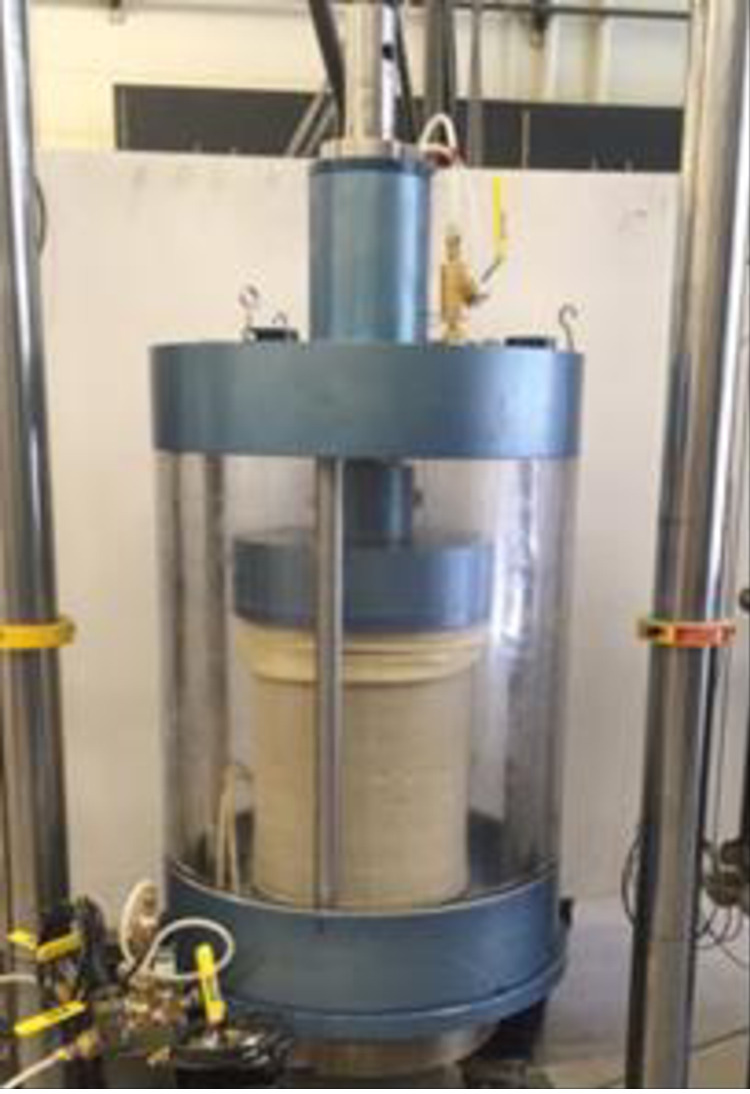
CHCT devices at UCD.

**Fig 4:**
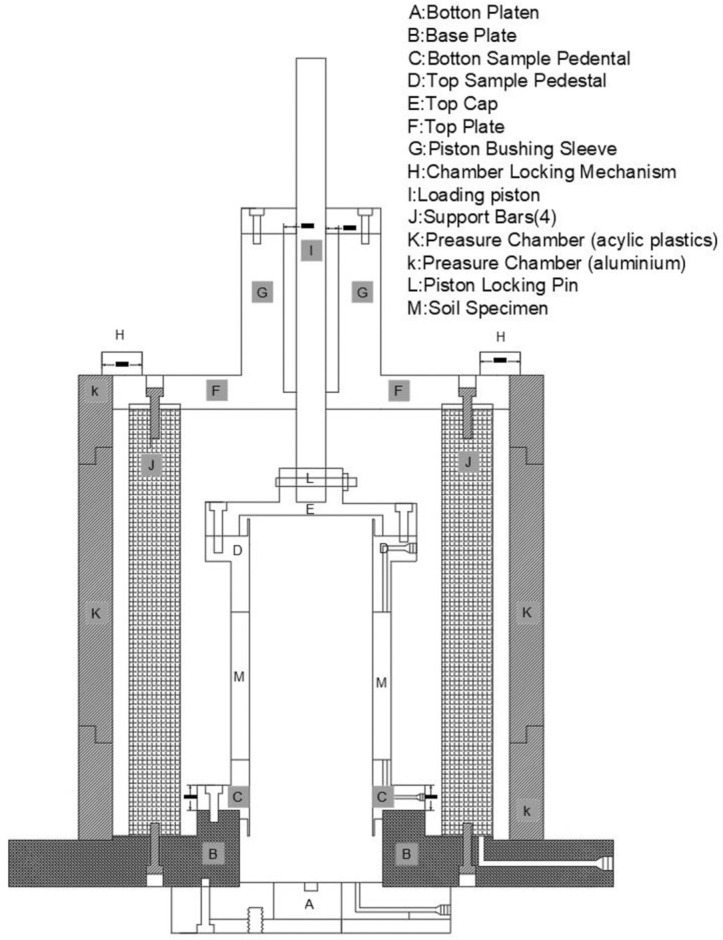
Schematic diagram of the cyclic hollow cylinder test device at UCD (J. W. Chen 1988 [21]).

The Hollow Cylinder Axial/Torsional cell, shown in Fig 3, is designed and fabricated based on the hollow cylinder cell initially created by Hight, D.W. [22] at Imperial College in 1983, incorporating further enhancements from J. W. Chen [23] in 1988 at UCD. A representation of the CHCT apparatus is shown in Fig 4.

To create a hollow cylindrical soil sample with a desired relative density, the sample is surrounded by both inner and outer membranes and positioned in a chamber. Equal confining pressures are then applied uniformly from both ends. Water is allowed to permeate upward from the bottom to help eliminate air bubbles. The back pressure, along with the confining (cell) pressure, is incrementally increased until the Skempton pore pressure parameter, B, reaches 0.95 or higher, ensuring that the sample is fully saturated. After saturation, the sample undergoes cyclic torsional loading at a chosen cyclic stress ratio with the INSTRON testing machine until it attains a state of liquefaction. This loading emulates a perfect simple shear scenario following the consolidation and saturation of the sample.

**Fig 5:**
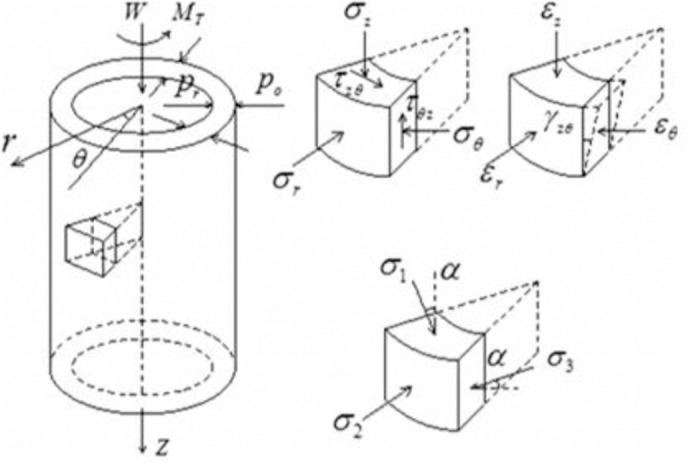
Loading and stress scenarios for hollow cylinder testing.

Cyclic torsional loading is applied using a rigid piston and top platen, while instruments gauge axial and rotational displacements and torsional and axial loads. The CHCT test subjected the sample to uniform inner and outer confining pressures and a constant axial load. Fig 5 shows the stress conditions within the hollow cylinder sample, while Table 4 summarizes the equations utilized to assess the strains and stresses in a CHCT sample.

**Table 4:** Equations governing stresses and strains in the CHCT (Hight, et al., (1983) [24]).

	Stress	Strain
Vertical	$\sigma_{z}=\frac{W}{\prod (r_{o}^{2}-r_{i}^{2})}+\frac{p_{o}r_{o}^{2}-p_{i}r_{i}^{2}}{\left(r_{o}^{2}-r_{i}^{2}\right)}$	$\varepsilon_{z}=\frac{z}{H}$
Radial	$\sigma_{r}=\frac{p_{o}r_{o}+p_{i}r_{i}}{r_{o}+r_{i}}$	$E_{r}=-\frac{u_{o}-u_{i}}{r_{o}-r_{i}}$
Circumferential	$\sigma_{\theta }=\frac{p_{o}r_{o}-p_{i}r_{i}}{r_{o}+r_{i}}$	$\varepsilon_{\theta }=-\frac{u_{o}+u_{i}}{r_{o}+r_{i}}$
Shear	$\tau_{\theta z}=\frac{3M_{T}}{2\Pi (r_{o}^{3}-r_{i}^{3})}$	$\gamma_{\theta z}=\frac{\theta (r_{o}^{3}-r_{i}^{3})}{2H(r_{o}^{2}-r_{i}^{2})}$
Major principal	$\sigma_{1}=\frac{\sigma_{z}+\sigma_{\theta }}{2}+\sqrt{{\left(\frac{\sigma_{z}-\sigma_{\theta }}{2}\right)}^{2}+\tau_{\theta z}^{2}}$	$\varepsilon_{1}=\frac{\varepsilon_{z}+\varepsilon_{\theta }}{2}+\sqrt{{\left(\frac{\varepsilon_{z}-\varepsilon_{\theta }}{2}\right)}^{2}+\gamma_{\theta z}^{2}}$
Intermediate principal	$\sigma_{2}=\sigma_{r}$	$\varepsilon_{2}=\varepsilon_{r}$
Minor principal	$\sigma_{3}=\frac{\sigma_{z}+\sigma_{\theta }}{2}-\sqrt{{\left(\frac{\sigma_{z}-\sigma_{\theta }}{2}\right)}^{2}+\tau_{\theta Z}^{2}}$	$\varepsilon_{3}=\frac{\varepsilon_{Z}+\varepsilon_{\theta }}{2}-\sqrt{{\left(\frac{\varepsilon_{Z}-\varepsilon_{\theta }}{2}\right)}^{2}+\gamma_{\theta Z}^{2}}$

## 5. Test results

This research conducts 37 CHCTs on soils with varying fine content to examine how fine materials influence soil liquefaction resistance. Each sample has an inner diameter of 203 mm, an outer diameter of 254 mm, and a height of 254 mm. The soil samples are categorized by three relative densities: 30%, 45%, and 60%.

Each sample consists of Monterey No. 0/30 Sand blended with various fine content percentages: 5%, 10%, 15%, 25%, and 35%. This sand has a plasticity index of 20.

Table 5 provides a summary of the characteristics, testing conditions, and outcomes of the sample mixtures. This paper provides a series of findings from the CHCT tests, emphasizing the relationship between the number of cycles, torques, and the excess pore pressure that contributes to liquefaction.

**Table 5:** Characteristics of soil sample mixtures, the test conditions, and the results are summarized.

Test No.	Fines content (%)	Target Relative Density (%)	Effective stress (KPa)	Cyclic shear stress (KPa)	Cyclic stress ratio	Number of Cycles
1	0	30	103.42	25.86	0.25	5
2	0	30	103.42	41.37	0.4	3
3	0	30	206.84	41.37	0.2	30
4	0	30	206.84	62.05	0.3	16
5	0	30	206.84	82.74	0.4	10
6	0	45	103.42	20.68	0.2	28
7	0	45	103.42	31.03	0.3	15
8	0	45	103.42	41.37	0.4	8
9	0	45	206.84	41.37	0.2	160
10	0	45	206.84	62.05	0.3	64
11	0	45	206.84	82.74	0.4	28
12	0	60	103.42	20.68	0.2	68
13	0	60	103.42	31.03	0.3	28
14	0	60	103.42	41.37	0.4	12
15	0	60	206.84	41.37	0.2	643
16	0	60	206.84	62.05	0.3	181
17	0	60	206.84	82.74	0.4	53
18	5	30	103.42	41.37	0.4	8
19	5	30	206.84	82.74	0.4	28
20	5	60	103.42	41.37	0.4	19
21	5	60	206.84	82.74	0.4	40
22	10	30	103.42	31.03	0.3	7
23	10	30	206.84	62.05	0.3	26
24	10	60	103.42	31.03	0.3	18
25	10	60	206.84	62.05	0.3	39
26	15	30	103.42	20.68	0.2	7
27	15	30	206.84	41.37	0.2	26
28	15	60	103.42	20.68	0.2	18
29	15	60	206.84	41.37	0.2	38
30	25	30	103.42	31.03	0.3	7
31	25	30	206.84	62.05	0.3	26
32	25	60	103.42	31.03	0.3	19
33	25	60	206.84	62.05	0.3	38
34	35	30	103.42	41.37	0.4	8
35	35	30	206.84	82.74	0.4	27
36	35	60	103.42	41.37	0.4	18
37	35	60	206.84	82.74	0.4	39

## 6. Test results

### 6.1 Cyclic shear stress versus the number of cycles to liquefaction

**Fig 6:**
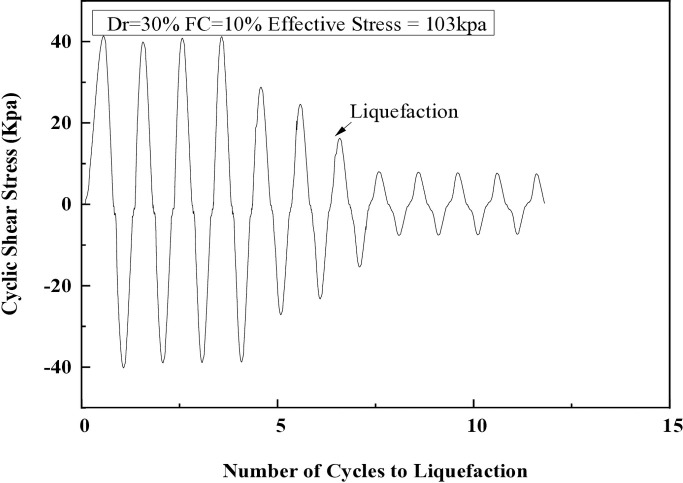
Cyclic shear stress versus the number of cycles to liquefaction in cyclic hollow cylinder test.

**Fig 7:**
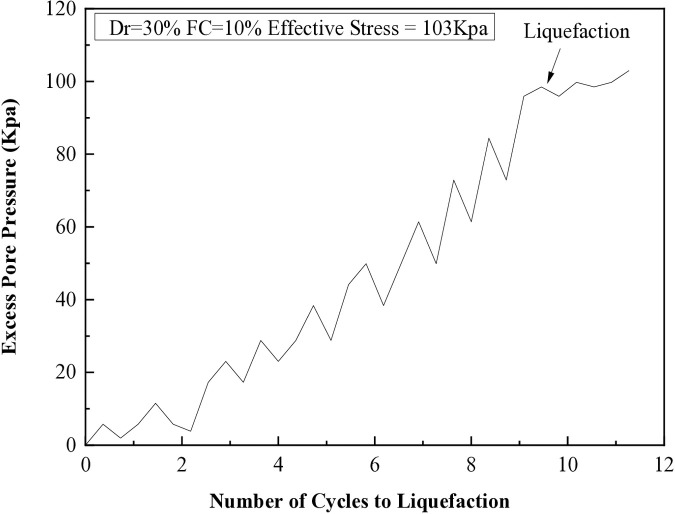
Change in pore water pressure in relation to number of cycles to liquefaction in the CHCT.

In a series of CHC tests, a constant cyclic shear stress of 41.37 kPa was applied to a saturated soil sample with 5% fines at 0.5 Hz. As illustrated in Fig 6, there was no measurable deformation of the specimen during the first four loading cycles, and the load amplitude remained stable. However, changes began to occur after the fourth cycle, with the shear stress amplitude decreasing to 27.58 kPa by the fifth cycle. By the seventh cycle, this dropped further to 13.79 kPa as the sample experienced liquefaction, with excess pore pressure reaching 15 psi and effective stress reducing to zero. Fig 7 shows that at this point, the axial strain reached + /- 10%, indicating liquefaction of the sample.

### 6.2 Excess pore pressure versus number of cycles to liquefaction

Fig 7 illustrates the relationship between excess pore pressure and the number of cycles during CHCT tests. The excess pore water pressure gradually increases during the initial four loading cycles, while sample shows no noticeable deformation. By the seventh cycle,excess pore pressure rises quickly, ultimately equaling the effective stress that is applied externally.

**Fig 8:**
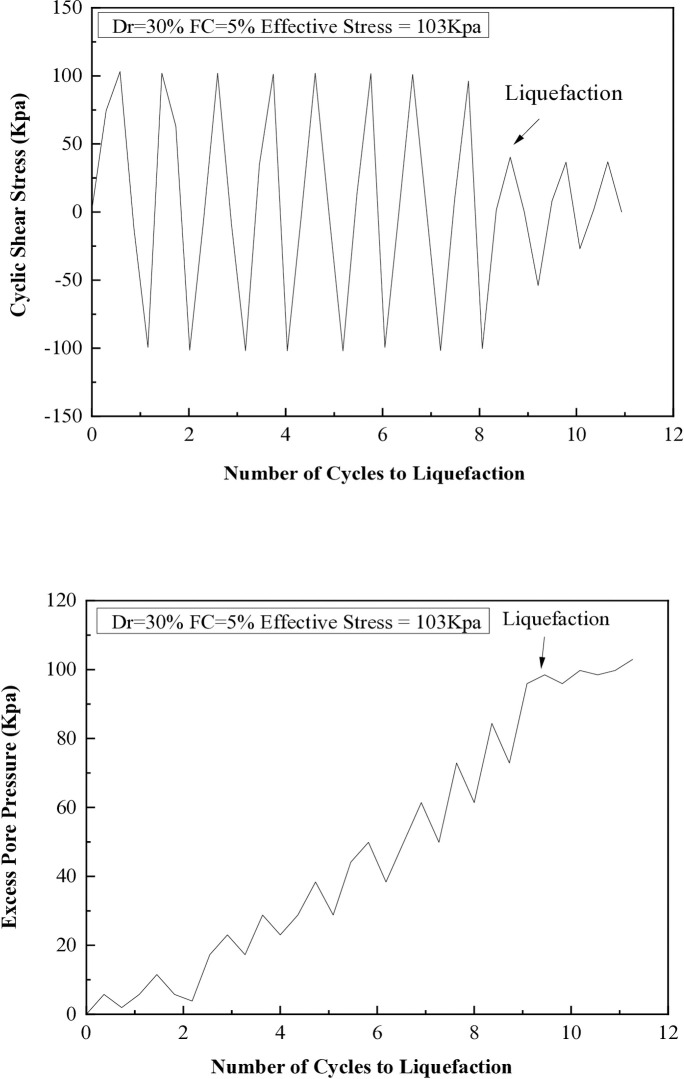
Cyclic shear stress and excess pore pressure versus number of cycles to reach liquefaction on soil sample (Dr=30%) with 5% of fine content.

**Fig 9:**
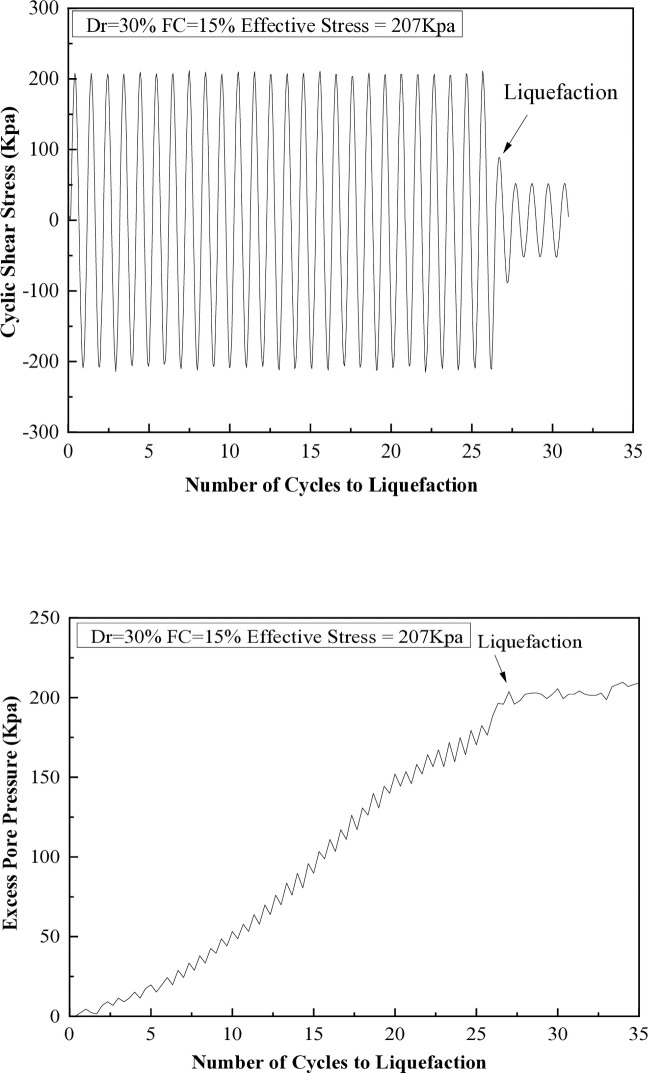
Cyclic shear stress and excess pore pressure versus number of cycles to reach liquefaction on soil sample (Dr=30%) with 15% of fine content.

**Fig 10:**
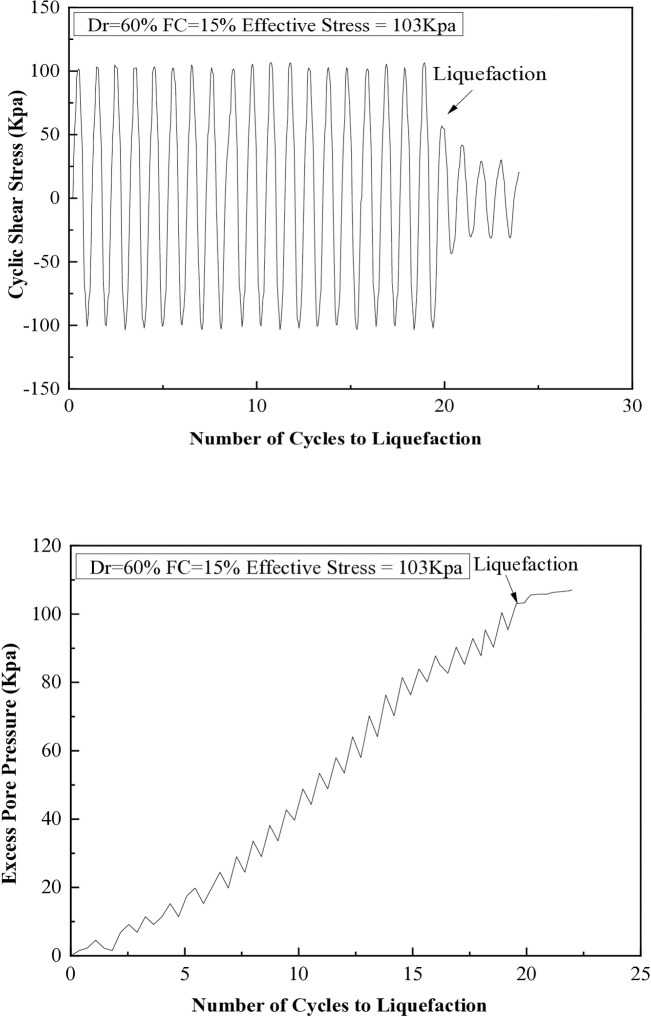
Cyclic shear stress and excess pore pressure versus number of cycles to reach liquefaction on soil sample (Dr=60%) with 15% of fine content.

**Fig 11:**
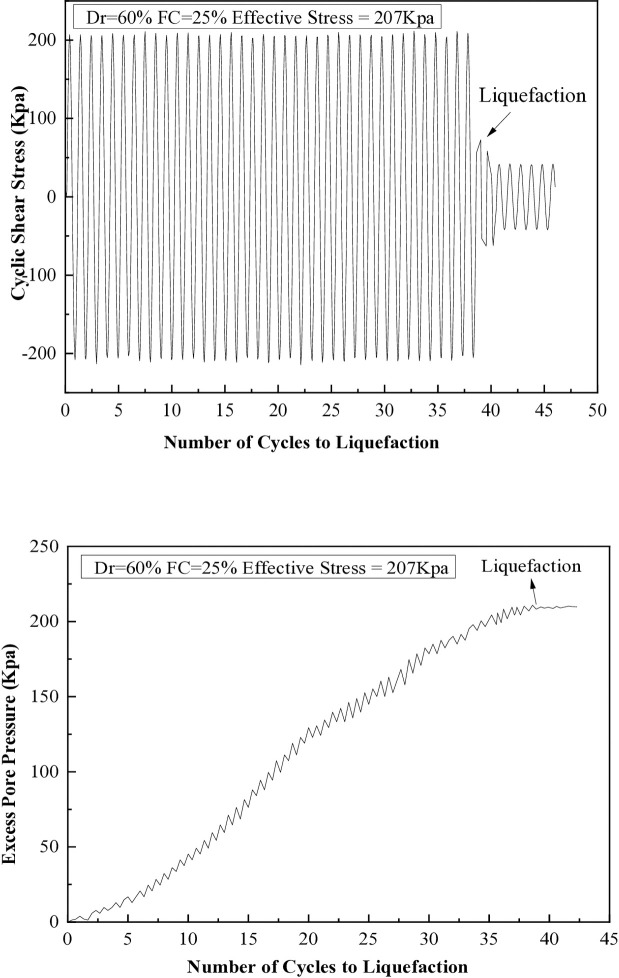
Cyclic shear stress and excess pore pressure versus number of cycles to reach liquefaction on soil sample (Dr=60%) with 25% of fine content.

**Fig 12:**
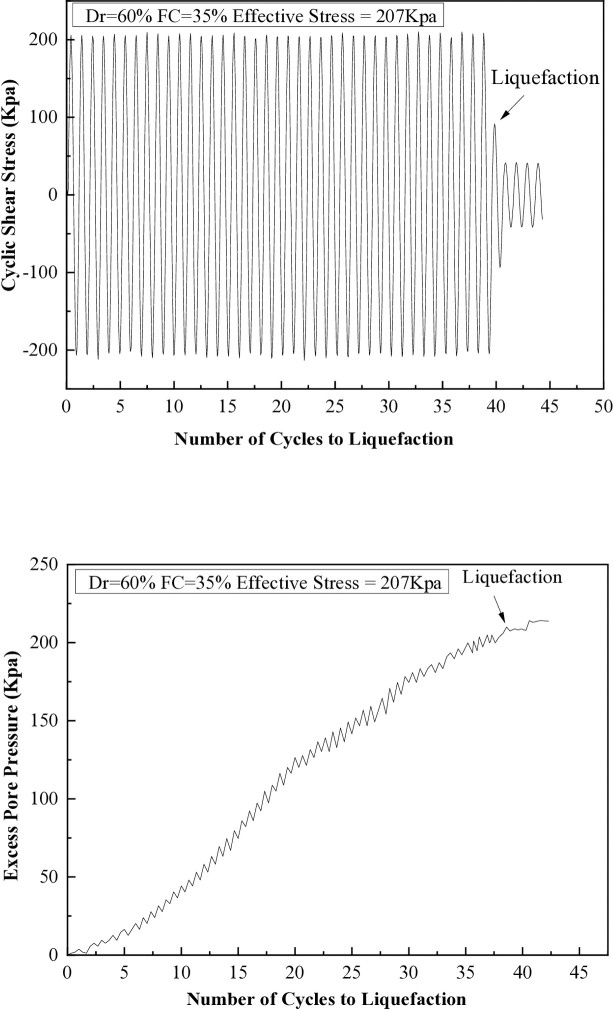
Cyclic shear stress and excess pore pressure versus number of cycles to reach liquefaction on soil sample (Dr=60%) with 35% of fine content.

It has been observed that when the excess pore pressure increases to 15 psi, signs of soil liquefaction are evident after the seventh cycle. The liquefaction resistances of soil samples with different fine content percentages (5%, 10%, 15%, 25%, and 35%) are illustrated in Figs 8 through 12. These figures show the relationship between excess pore pressure and the number of cycles that result in liquefaction Figs 9–12.

### 6.3 Stress ratio versus number of cycles to liquefaction

**Fig 13:**
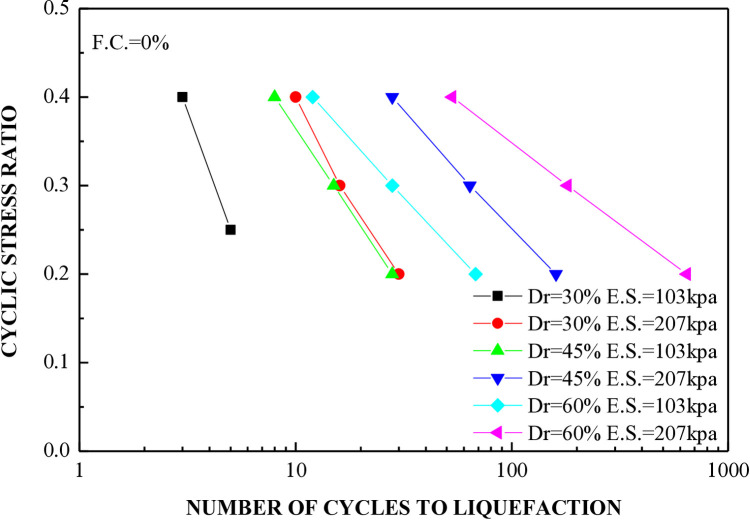
Cyclic stress ratio versus number of cycles to reach liquefaction for clean sand under effective stress of 103kpa and 207kpa.

Fig 13 shows the relationship between the stress ratio and number of cycles leading to liquefaction in soil of different densities while keeping the fine content and plasticity index constant. A series of seventeen CHCTs were conducted using uniform medium clean sand. The sand samples were adjusted to relative densities of 30%, 45%, and 60%, and were then tested under confining pressures of 103 kPa and 207 kPa. Fig 13 illustrates that the number of cycles required for liquefaction rises as the relative density increases. The sample exhibiting 30% relative density, tested under the same cyclic stress ratio with an effective mean stress of 103 kPa, experiences liquefaction after fewer cycles.

**Fig 14:**
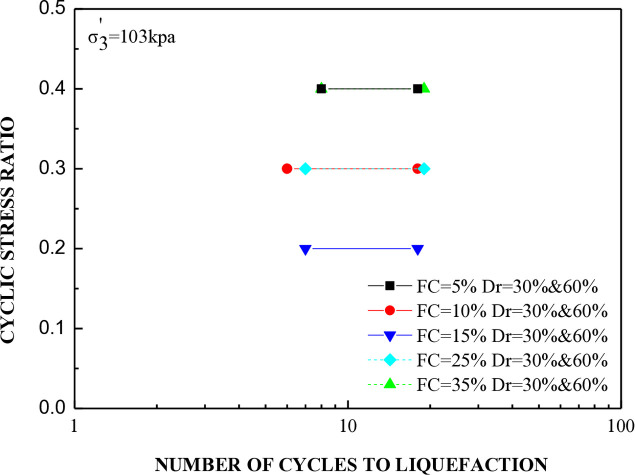
Cyclic stress ratio versus number of cycles to reach liquefaction for soil specimens with varying percentages of fine content under effective stress of 103kpa.

**Fig 15:**
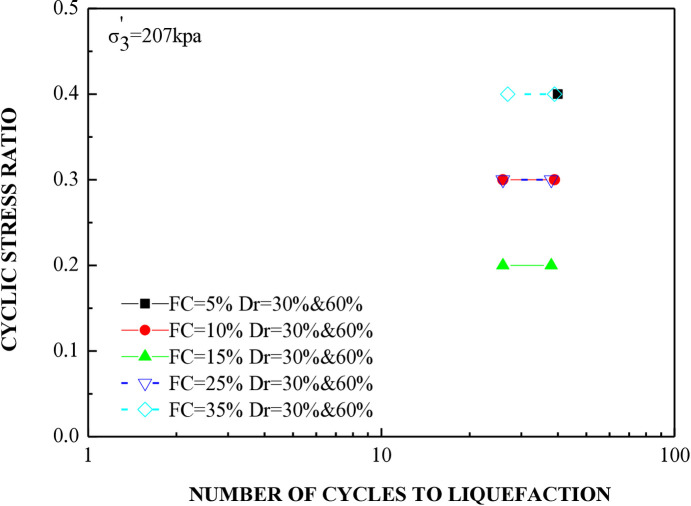
Cyclic stress ratio versus number of cycles to reach soil liquefaction for soil specimens with varying percentages of fine content under effective stress of 207 kpa.

The cyclic stress ratio diminishes linearly as the number of liquefaction cycles rises. Soil samples with different relative densities demonstrate that denser soils tend to undergo liquefaction after a greater number of cycles. The sample consolidated at a higher confining pressure liquefies at a higher loading cycle. A total of 20 cyclic hollow cylinder tests were conducted, as illustrated in Figs 14 and 15. The tests utilized uniform medium sand with a plasticity index (P.I.) of 20%. Fines content was varied at 5%, 10%, 15%, 25%, and 35%.The soil specimens were produced at relative densities of 30%, 45%, and 60% and were evaluated under effective stresses of 103 kPa and 207 kPa.

Figs 14 and 15 illustrate that the number of cycles required for liquefaction remains constant regardless of the increase in fine content at the same stress ratio. It was observed that soil with a 15% fines content, when prepared at a relative density of 30% and subjected to effective stresses of 103 kPa and 207 kPa, displayed the lowest stress ratio while undergoing an equivalent number of cycles to liquefaction.

### 6.4 Stress ratio vs different percentages of fines contents for 8, 20, 27, and 40 cycles to liquefaction

This research investigates the cyclic stress ratio necessary to induce liquefaction after 8, 20, 27, and 40 cycles for all soil samples tested at 30% and 60% during the CHCT evaluation.

**Fig 16:**
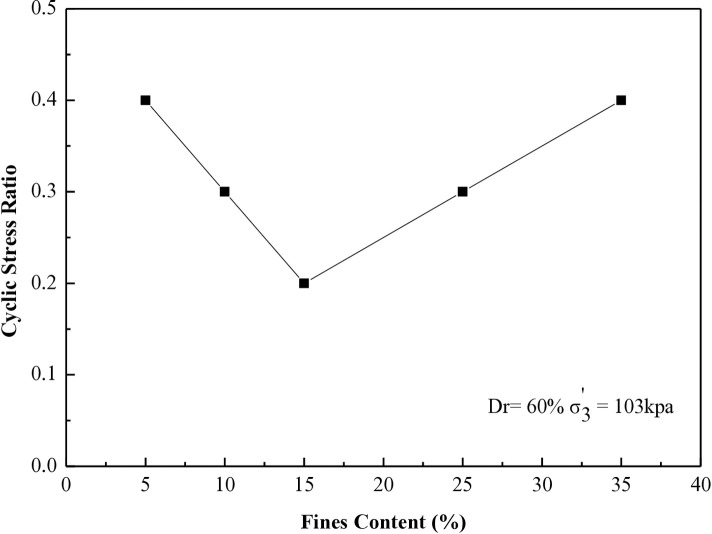
Cyclic stress ratio requires for reaching soil liquefaction after 8 cycles versus different soil specimens in CHCT.

**Fig 17:**
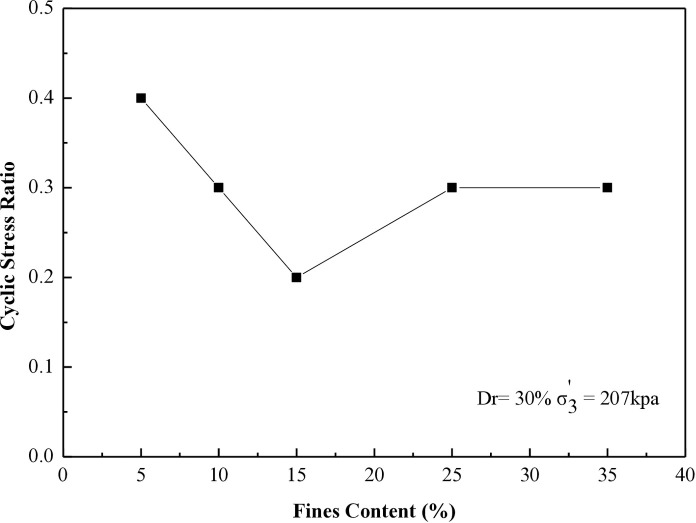
Cyclic stress ratio requires for reaching soil liquefaction after 27 cycles versus different soil specimens in CHCT.

Figs 16 and 17 display the outcomes from all specimens prepared at a relative density of 30%, emphasizing tests performed under effective stresses (σ_3_’) of 103 kPa and 207 kPa. Figure 16 illustrates the connection between the cyclic stress ratios needed to induce soil liquefaction after 8 cycles and soil samples containing five different fine content percentages.

Fig 17 illustrates the correlation between the cyclic stress ratios necessary for inducing soil liquefaction over 27 cycles and soil samples containing varying amounts of fines at five different percentages.

**Fig 18:**
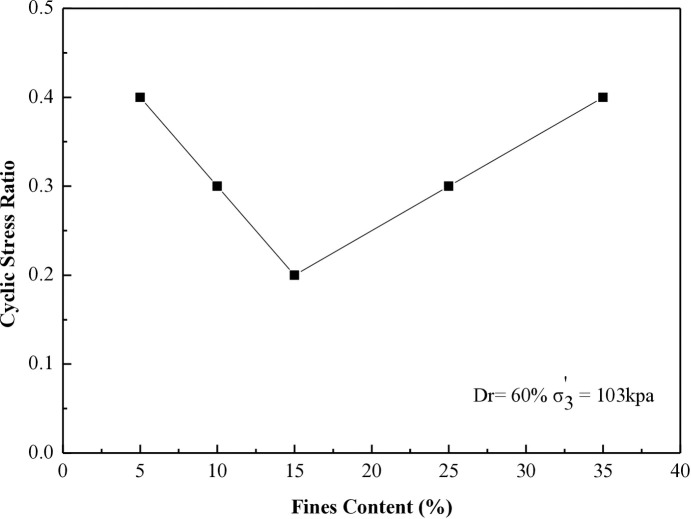
Cyclic stress ratio requires for reaching soil liquefaction after 20 cycles versus different soil specimens in CHCT.

**Fig 19:**
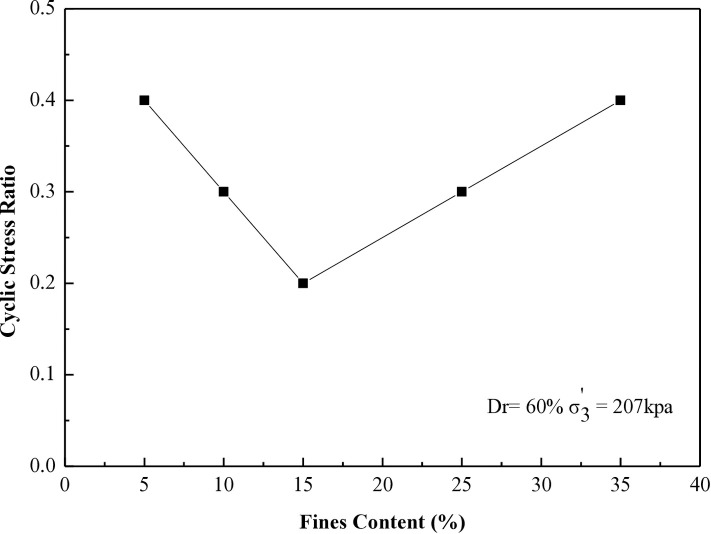
Cyclic stress ratio requires for reaching soil liquefaction after 40 cycles versus different soil specimens in CHCT.

Figs 18 and 19 demonstrate the relationship between the cyclic stress ratio and different soil specimens prepared at a relative density of 60%. The tests were conducted under effective stresses (σ_3_’) of 103 kPa and 207 kPa, with differing fine contents for 20 and 40 cycles, comparable to the data presented in Figs 16 and 17.

The resistance to liquefaction in soil diminishes with higher fine content until it hits 15%. Beyond 15%, the soil’s resistance to liquefaction improves as the fine content continues to increase. This paper establishes 15% as the critical threshold for fine content, as indicated by the results of the CHCT test.

## 7. Predicting cyclic stress ratio using back propagation neural network (BPNN)

**Fig 20:**
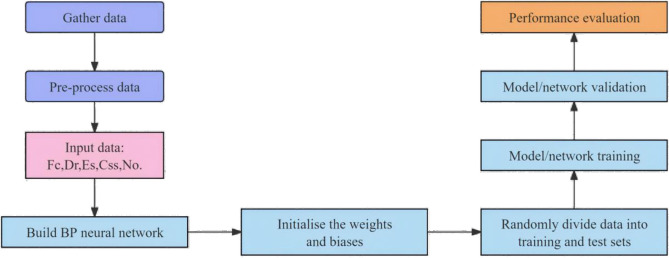
Flow chart of the machine learning model.

**Fig 21:**
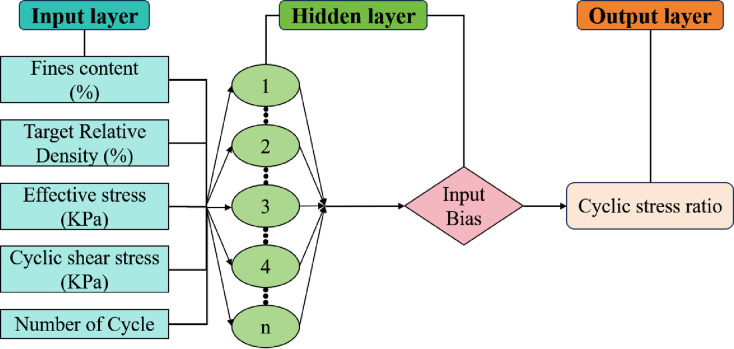
Schematic diagram of BPNN.

The flow chart of the machine learning model is shown in Fig 20, which employed BPNNs comprising input layer, hidden layers, and output layer modules, as shown in Fig 21. The input layer encompassed five parameters closely associated with cyclic stress ratio: cyclic shear stress (C_ss_ in kPa), fines content (F_c_ in %),Target Relative Density (Dr in %), Number of Cycles (No.) and effective stress (E_s_ in kPa).

The foundation of the BPNN model in this study was laid upon a comprehensive database consisting of 37 instances as presented in Table 5. The soil samples are categorized by three target relative densities: 30%, 45%, and 60%. Each sample consists of Monterey No. 0/30 Sand blended with various fine content percentages (F_c_): 0%, 5%, 10%, 15%, 25%, and 35%. Number of Cycles (No.) are ranged from 3 to 643. Cyclic shear stresses (C_ss_) are ranged from 20,68 kpa to 82.74kpa. Two effective stresses (E_s_) values of 103.42 kpa and 206.84 kpa are using to perform in this research.

The steps of neural network model outlined, such as repeated training, L_2_/dropout regularization, and early stopping while monitoring validation, work together to help the BPNN model achieve good generalization. Consequently, the model’s final performance is reliable and not merely a result of overfitting.

The model underwent multiple training sessions with varying initializations. Remarkably, all attempts yielded nearly identical results, indicating that the model’s performance is robust against random weight initialization.

To enhance the network’s generalization capabilities, L_2_ weight decay regularization and dropout layers were incorporated. These techniques discourage overly complex weights, prompting the network to focus on learning features that are more applicable to different datasets.

Additionally, early stopping was employed by monitoring the validation error (loss) throughout the training process. Training was paused whenever the validation loss stopped improving, which helps prevent the model from simply memorizing the training data.

**Fig 22:**
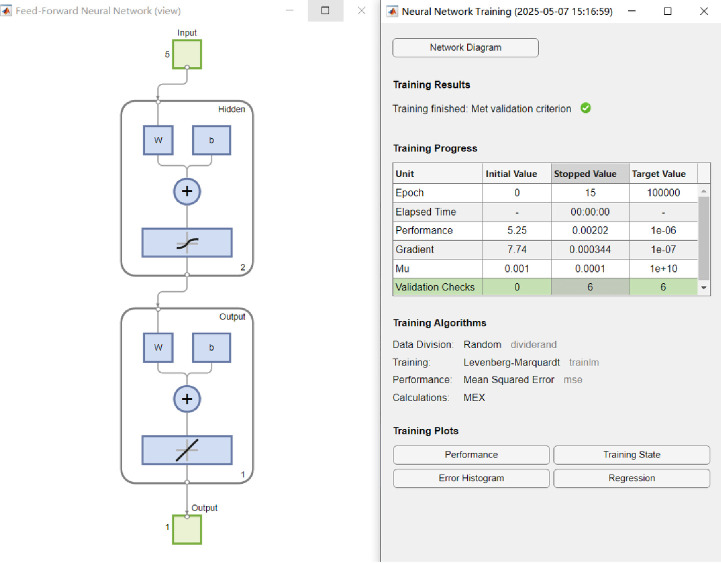
Neural network inside steps.

The data set was divided into a training set, consisting of 90% of the total data, and a validation set, comprising the remaining 10%. This division was conducted randomly while maintaining the overall distribution of the target variable to minimize any potential bias that might occur from a sequential split. The 90%/10% ratio was selected to ensure there was enough data for effective training while still retaining a sufficient amount for monitoring performance. To further validate the model’s consistency, this random splitting was executed multiple times. Additionally, 5-fold cross-validation was employed to enhance the reliability of the model’s evaluation. In this method, the data is partitioned into k equal segments or folds. The model is trained on k-1 of these folds and validated on the remaining one, a process that is repeated so that each fold is used as the validation set at least once. This technique ensures that every data point is utilized for both training and validation, providing a more thorough and reliable estimate of the model’s performance compared to a single train/validation split. Cross-validation helps reduce the impact of any single split and delivers a more consistent performance estimate, as confirmed by standard references. In this work, 90% of the data was allocated to the training set, while 10% was allocated to the validation set. The BPNN model was generated by MATLAB program, and the program page is shown in Fig 22.

**Fig 23:**
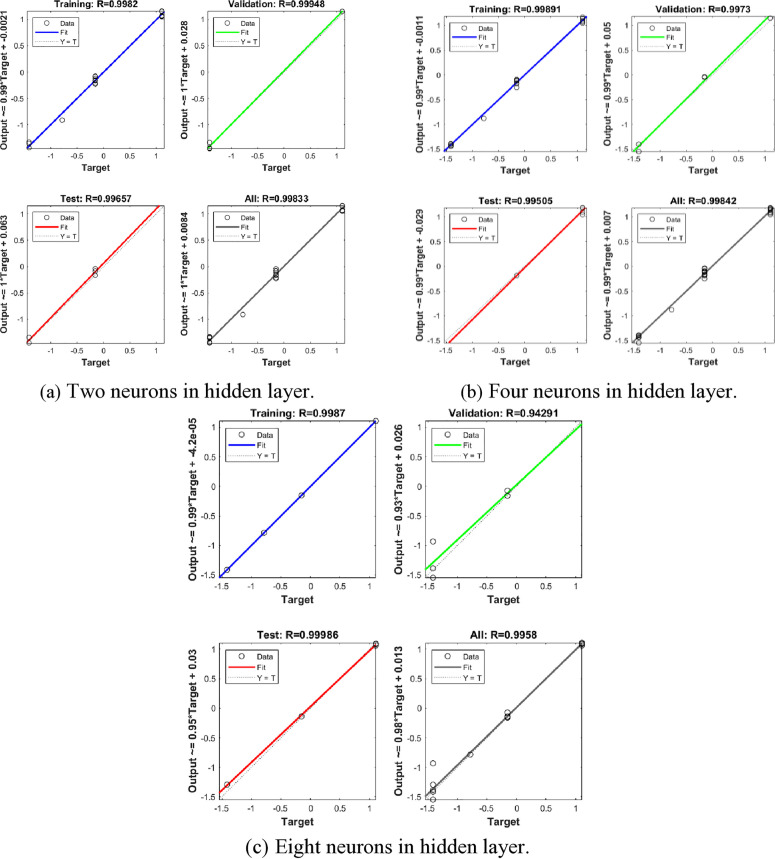
Effect of neuron numbers on the training model. (a) Two neurons in hidden layer.Four neurons in hidden layer. (b) Eight neurons in hidden layer.

For the purpose of model training, different numbers of neurons were investigated in this study, specifically 2, 4, and 8, the regression plots for the training, validation, test and all sets along with their R values are illustrated in Fig 23.

It was observed that employing two neurons in the hidden layer resulted in a highly accurate trained model that closely approxi- mated the true value, with R values approaching unity. Moreover, the R value of the test set was 0.99842 (Fig 23(b)), indicating that the developed BP neural network model performed well on unseen data. However, with an increase in the number of neurons from 4 to 8,the R values of validation set decreased from 0.99842 to 0.9958 due to over- fitting issues. Consequently, two neurons in hidden layer were applied.

**Fig 24:**
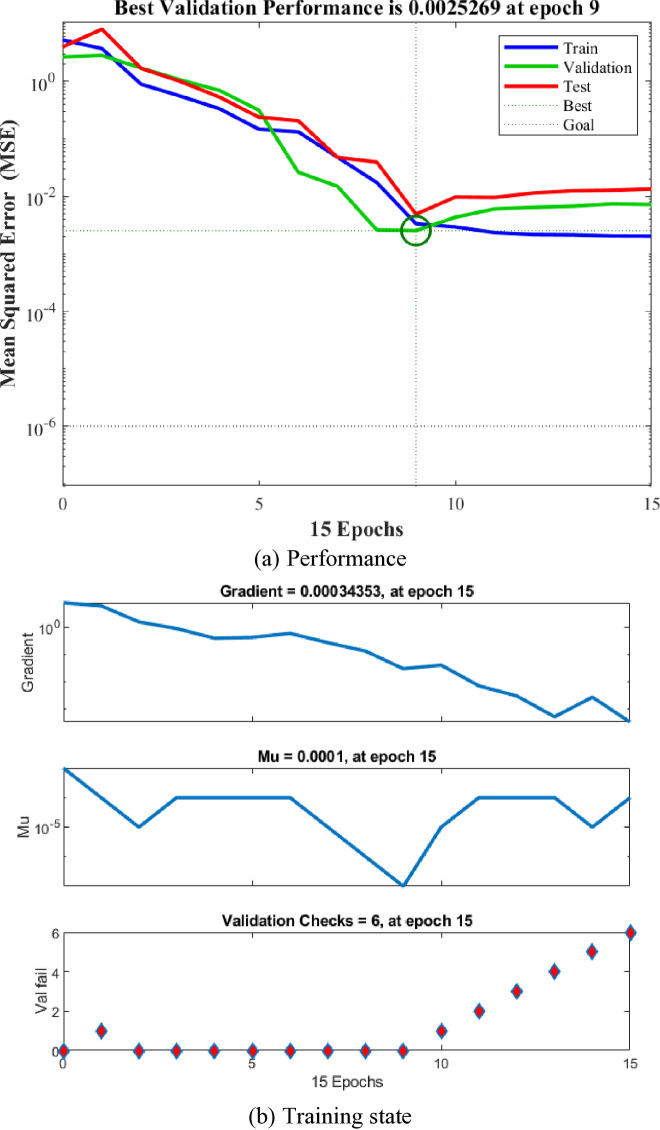
Validation performance of the BP neural network. (a) Performance. (b) Training state.

The validation performance plot of the BP neural network model in terms of mean squared error (MSE) is illustrated in Fig 24. It was evident that, both the training and validation sets displayed high MSE values at the initial training process. However, these values gradually decreased to lower levels, indicating successful learning of the relationship between inputs and outputs by the BPNN model. The best training performance was attained by the BPNN at the ninth epoch with a MSE of 0.0025269.

Figs 23 and 24 have been modified to illustrate the training and validation error curves throughout the epochs. These figures reveal that the training and validation errors converge closely, both trending down to a stable level. Specifically, Fig 23 shows the error curves for a typical run, while Fig 24 offers the averaged outcomes from several runs. In both instances, there is no significant increase in validation error as the training error declines, suggesting that overfitting was not an issue.

**Fig 25:**
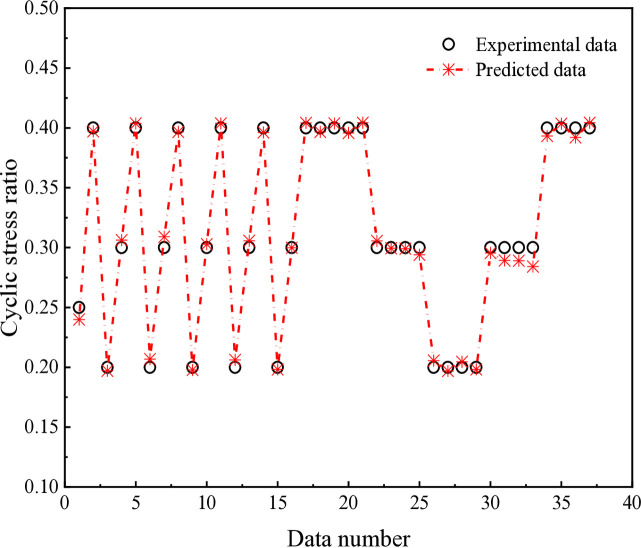
Comparison between predicted and actual values.


The predicted cyclic stress ratios leading to initial liquefaction after cyclic loading cycles are illustrated in Fig 25, demonstrating an excellent agreement with actual values. To evaluate performance of BPNN model, root mean squared error (RMSE) and mean absolute percentage error (MAPE) were computed as evaluation metrics in this study using the following expressions:

$$RMSE=\;\sqrt{\sum_{i=1}^{n}{\left(e_{i}-p_{i}\right)}^{2}/n}$$ (1)

$$MAPE=\;(\sum_{i=1}^{n}|e_{i}-p_{i}|/e_{i})/n$$ (2)

Where n represent the total numbers of the samples, e_i_ and p_i_ denote the actual and predicted outputs, respectively.

It is noteworthy that any developed or proposed model is only applicable within the input parameter defined by the limitation of the database numbers. This limitation emphasizes the significance of ensuring that the input data for practical applications fall within the specified parameter boundaries. Besides, the developed BPNN model in this work is primarily focused on predicting cyclic stress ratio (one neuron in output layer) leading to initial liquefaction after cyclic loading cycles, without considering predictions of the complete testing results curves.

Given the valuable information provided by the testing results curve, including cyclic shear stress versus number of cycles to liquefaction, excess pore pressure versus number of cycles to liquefaction and so on. It is necessary to integrate BP neural network and random forest algorithms for predicting the complete curve of testing results in further research.

## 8. Discussion

This study examines the effect of fine material content on soil resistance to liquefaction by executing 37 isotopically consolidated undrained CHCTs. The findings suggest a critical fine content threshold, which acts as a key point on the curve connecting stress ratio to fine content and differentiating between two categories of mixtures. Below this threshold, increasing fine content weakens liquefaction resistance, while above it, higher fine content enhances resistance. This threshold fine content was identified as 15% for the soils tested.

Results from the cyclic hollow cylinder tests indicate that soil liquefaction resistance diminishes as fine content rises, reaching its lowest resistance around the 15% level. However, historical case studies suggest that cyclic stress ratios can increase with higher fines content when the modified standard penetration blows count remains constant. To accurately assess the liquefaction potential of soils, it’s essential to consider the stress ratio (SR) of liquefaction resistance in clean sands compared to soils with fines below their threshold, typically under 20%.

Consequently, it is crucial to revise current methods for evaluating soil liquefaction resistance in fine-containing soils. This study proposes the BPNN model gives more accurated predictions on cyclic stress ratios leading to initial liquefaction after cyclic loading cycles, and also assess liquefaction potential of soil samples by incorporating factors such as cyclic shear stress (Css in kPa), fines content (Fc in %),Target Relative Density (Dr in %), Number of Cycles (No.) and effective stress (Es in kPa).

By leveraging the results from the thirty-seven CHCT tests, these models aim to better mimic the cyclic stresses experienced in the field during earthquakes, utilizing samples with five distinct fine contents and three relative densities.

While laboratory-reconstituted samples may differ from field samples, these models provide reliable predictions of cyclic stress ratios that lead to initial liquefaction within 10 and 30 cycles of loading. The proposed models and the determined threshold fine content serve as important resources for engineers, allowing for a more precise evaluation of liquefaction resistance in soils containing fines.

## 9. Conclusion

This study offers an extensive summary of the current approaches for assessing liquefaction resistance and analyzes the factors that influence it. Furthermore, this study performed 37 isotopically consolidated undrained CHCTs to examine how the fine content affects soil liquefaction resistance.

For the experiments, Monterey No. 0/30 Sand was mixed with LC to create samples with varying levels of fine content: 5%, 10%, 15%, 25%, and 35%. The fine mixture utilized in this study had a plasticity index of 20%. BPNN model was developed to predictcyclic stress ratio leading to initial liquefaction after cyclic loading cycles.

The main conclusions drawn from this research are as follows:

1. At two constant relative densities of 30% and 60%, an increase in fine content up to 15% leads to a reduction in liquefaction resistance compared to the clean parent sand. However, beyond 15% fine content, further increases result in enhanced liquefaction resistance.
2. A certain threshold of fine content is anticipated, signifying a transition from sand-dominated traits to those influenced more by finer particles.This threshold is estimated to be around 15% in the soils examined.
3. Several factors influence the resistance to liquefaction, including the number of cycles causing liquefaction, relative density, fines content, and the confining pressure.
4. The applicability of existing codes provisions and models was evaluated through an experimental database. To enhance accuracy, a BPNN model was developed for predicting cyclic stress ratio leading to initial liquefaction after cyclic loading cycles. The findings demonstrated that this BPNN model exhibited superior precision and efficiency compared to conventional models, with MAPE values of 1.05%, respectively.
